# Evaluation of the Low Inclusion of Full-Fatted *Hermetia illucens* Larvae Meal for Layer Chickens: Growth Performance, Nutrient Digestibility, and Gut Health

**DOI:** 10.3389/fvets.2020.585843

**Published:** 2020-11-27

**Authors:** Xiaohua Chu, Mengmeng Li, Guiying Wang, Kuiming Wang, Rongsheng Shang, Ziyu Wang, Lusheng Li

**Affiliations:** College of Agronomy, College of Mechanical & Automotive Engineering, College of Life Science, Liaocheng University, Liaocheng, China

**Keywords:** full-fatted black soldier fly larvae meal, layer chicken, growth performance, gut health, antioxidant ability

## Abstract

Substitution of feed protein source with defatted black soldier fly larvae meal (BSFM) has been evaluated intensively in poultry, but information about full-fatted BSFM is still very limited. The aim of the present study was to investigate the effect of dietary low inclusion of full-fatted BSFM on the growth performance, plasma antioxidant ability, nutrient digestibility, and gut health of layer chickens during 1–42 days of age. A total of 480 female 1-day-old Hy-Line Brown chickens were divided into four dietary treatments, with the inclusion of 0, 3, 6, and 9% of full-fatted BSFM. Each treatment included six replicates and 20 birds per replicate. As dietary full-fatted BSFM inclusion levels increased, there was a quadratic increase in final weight and average daily gain and a quadratic decrease in feed/gain ratio. Dietary full-fatted BSFM inclusion levels increased the digestibility of crude protein and ether extract quadratically as well as ileum mucosal sIgA concentration linearly, but these had no effect on intestinal morphology. Additionally, an increase in dietary full-fatted BSFM inclusion levels resulted in a linear increase in glutathione peroxidase and total superoxide dismutase activities and a linear decrease in malondialdehyde content in plasma. The encouraging results of the improvement of growth performance, nutrient digestibility, antioxidant ability, and gut health parameters suggested that partially full-fatted BSFM inclusion can be suitable protein ingredients for layer chickens' diets at the starter period.

## Introduction

Due to the increasing demand of feed protein sources and environment-friendly production, insects have received considerable attention as alternative protein sources to replace conventional protein sources used in animal production (1, 2). The nutritional components in black soldier fly larvae meal (BSFM), characterized by balanced amino acid composition and a large amount of mono- and poly-unsaturated fatty acids, have been the most appealing for the feed industry (3). The potential use of BSFM as a promising insect meal has been well-studied to evaluate the possibility of substitution for soybean meal in hens (4, 5), broilers (6), and ducks (7). So far, the utilization of BSFM has been evaluated in layer hens at the middle and the late laying periods (8), but limited knowledge is available for layer chickens at the starter period. Therefore, more efforts are still required to ensure the scientific and rational use of BSFM for poultry species at different growth phases. In addition, most studies have focused on the potential use of defatted BSFM in feed formulation of poultry (4, 6, 9, 10), whereas information about full-fatted BSFM is still very limited. However, high-level addition of full-fatted BSFM in the diet could cause oxidative rancidity of feed due to the high content of fat and unsaturated fatty acid. Previous studies have demonstrated that the dietary low inclusion of full-fatted BSFM (11, 12) or BSFM fat as an alternative to soybean oil (13) has positive effects on energy availability, attributed to improving growth performance and meat quality in species of pig and fish. These encouraging results suggested that there would be a promising practical application of the low inclusion of full-fatted BSFM in the formulation of poultry diets.

In the recent year, consumers' pressure and worries toward the harmful effects of antibiotic use have prompted researchers to receive considerable attention concerning gut health in poultry production (14). In terms of BSFM, a proportion of lauric acid (15) and antioxidant peptide (16) was characterized by enhancing the antioxidant ability and the antibacterial activity. For example, lauric acid could enhance intestinal immune ability due to their antioxidant and prebiotic properties and bacteriostatic effects on Gram-negative bacteria (17). Up to now, full-fatted BSFM has been evaluated to estimate the quantities of replacement required to sustain growth and improve meat quality (11, 12). Only few reports on full-fatted BSFM are available for gut health regarding nutrient digestibility, intestinal morphology, and immune status in poultry. The starter brooding period is critical for the development of growth, bone, and gastrointestinal tract to minimize mortality and keep the uniformity of young laying hens. In addition, the well-developed growth and gut in layer chickens at the starter brooding period directly affect the later egg production performance of laying hens. Thus, it is necessary to explore the effect of full-fatted BSFM on the gut health of layer chickens at the starter brooding period under the condition of the ban of antibiotics in China. In the present study, the effect of dietary inclusion of full-fatted BSFM on growth performance, plasma antioxidant ability, nutrient digestibility, and gut health has been investigated in layer chickens from 1 to 42 days of age.

## Materials and Methods

### Source of Full-Fatted BSFM

Black soldier fly larvae was bred and collected from a commercial company (Shandong Wooneng Agricultural Science and Technology Co., Ltd., Liaocheng, China). After hatching, BSF larvae were put on a mix of flour (35%) and water (65%) for 5 days. Then, the larvae were reared in a substrate of vegetable waste from 5 to 15 days of age. Afterward, full-fatted BSFM was prepared by the microwave drying method as described previously (18) and stored until needed for chemical analysis and diet formulation. The nutrient composition of full-fatted BSFM is as follows: dry matter (DM), 92.22%; crude protein (CP), 34.97%; ether extract (EE), 35.49%; Ca, 4.39%; P, 0.83%; chitin, 4.65%; dl-methionine, 0.50%; and l-lysine, 2.10%.

### Birds and Experimental Design

The experimental protocol was reviewed and approved by the Animal Care and Use Committee of Liaocheng University. A total of 480 female 1-day-old Hy-Line Brown layer chickens were divided into four treatments, with six replicates per treatment and 20 birds in each replicate, and housed from 1 to 42 days of age. The corn–soybean meal basal diet which served as the control diet without full-fatted BSFM inclusion was formulated to satisfy the basic nutrient requirements recommended (Metabolizable energy, 12.6 MJ/kg, Crude protein, 18%) by the NRC (1994) for layer chickens at the starter phase. The other three dietary treatments were formulated with increasing levels of 3, 6, and 9% full-fatted BSFM as a partial substitution for conventional protein/fat sources in the basal diet. The composition and the nutrient levels of four experimental diets are listed in Table 1. All experimental diets were iso-caloric and isonitrogenous and contained TiO_2_ as digestibility marker. The birds were allowed free access to feed and water during the experimental period. The room was equipped with an intelligent light control device to ensure 12 h of light and 12 h of dark per day. The temperature inside the building was 32–33°C on the first week and was reduced by 2–3°C each week. On day 28, the temperature was set at 21°C until the end of the experiment. Feed consumption and mortality were recorded for each replicate pen. At 42 days of age, after 12 h of fasting, the birds were weighed, and the average daily gain (ADG), average daily feed intake (ADFI), and feed/gain ratio (F/G) were calculated and corrected for mortality. To detect nutrient digestibility, fecal samples were collected from each pen from days 40 to 42 during the feeding experimental period. Feces collected each day from each pen was mixed to prepare composite samples and then dried at 65°C for 72 h before they were crushed and sieved through a 1-mm screen.

**Table 1:** Composition and nutrient levels of experimental diets (as fed basis).

**Items (%)**	**Inclusion levels, %**			
	**0**	**3**	**6**	**9**
**INGREDIENTS**				
Corn	66.76	67.58	65.41	66.06
Soybean meal	26.25	24.26	22.77	20.23
Corn gluten meal	2.00	1.36	1.00	0.00
Full-fatted black soldier fly larvae meal	0.00	3.00	6.00	9.00
Soybean oil	1.16	0.82	0.56	0.45
Limestone	1.13	1.29	1.45	1.45
Dicalcium phosphate	1.49	1.48	1.70	1.70
l-lysine·HCl	0.10	0.10	0.00	0.00
dl-methionine	0.11	0.11	0.11	0.11
Vitamin and mineral premix^a^	1.00	1.00	1.00	1.00
Total	100.0	100.0	100.0	100.0
**NUTRIENT LEVELS^b^**				
Metabolizable energy, MJ/kg	12.60	12.60	12.60	12.60
Crude protein, %	19.93	19.85	20.06	19.97
Crude fat, %	3.61	4.21	4.80	5.60
Calcium, %	0.92	1.07	1.01	0.96
Non-phytate phosphorus, %	0.40	0.42	0.45	0.39
Lysine, %	1.15	1.15	1.15	1.15
Methionine, %	0.44	0.44	0.44	0.44

a *Provided per kilogram of diet: vitamin A, 117,000 IU; vitamin D_3_, 3,600 IU; vitamin E (all-rac-tocopherol-acetate), 21 IU; vitamin B_1_, 3 mg; vitamin B_2_, 10.2 mg; vitamin B6, 5.4 mg; vitamin B_12_, 0.024 mg; folate acid, 0.9 mg; d-pantothenic acid, 15 mg; nicotinic acid, 45 mg; biotin, 0.15 mg; Fe (FeSO_4_·7H_2_O), 40 mg; Cu (CuSO_4_·5H_2_O), 6.8 mg; Zn (ZnSO_4_), 83 mg; Mn (MnSO_4_·H_2_O), 80 mg; Se (Na_2_SeO_3_), 0.30 mg; I (KI), 1.0 mg*.

b *Crude protein, crude fat, calcium, and non-phytate phosphorus were measured values; metabolizable energy, lysine, and methionine levels were calculated values*.

### Sample Preparation and Analysis

Nutrient analyses of the full-fatted BSFM and the experimental diets were carried out in duplicate. The DM, CP, EE, ash, and Ca contents of the selected samples were determined according to AOAC (19) using the 934.01, 976.05, 920.39, 942.05, and 984.27 methods, respectively. Dietary non-phytate phosphorus was determined as described by Liu et al. (20). The TiO_2_ content was measured on a UV spectrophotometer (Unican UV–vis Spectrometry, Helios Gamma, UK). The ash-free ADF and the residual nitrogen in ADF [ADFN; method 973.18; AOAC (19)] were determined and used to estimate the amount of chitin according to Marono et al. (21): chitin (%) = ash-free ADF (%) – ADFN (%). Amino acid contents in the full-fatted BSFM were analyzed using an amino acid analyzer (model L-8500A, Hitachi Ltd., Chyoudaku, Japan). The total tract-apparent digestibility (TTAD) of nutrients was calculated based on the concentration of TiO_2_ as an external marker in the diet and feces and according to the following formula: TTAD (%) = 100 – [(DTiO_2_/FTiO_2_) × (FN/DN) × 100], where TTAD is the total tract-apparent nutrient digestibility of DM, CP, OM, and crude fat, DTiO_2_ is the concentration of acid-insoluble ash in the diet; FTiO_2_ is the concentration of TiO_2_ in the feces, FN is the concentration of nutrients in feces, and DN is the concentration of nutrient in the diet.

Blood samples were collected *via* a bronchial vein from two fasted birds from each pen according to the average body weight (BW) within each replicate pen. Plasma samples were obtained by centrifuging blood samples at 3,000 × *g* for 20 min at 4°C and stored at 20°C for further analyses. Total antioxidant capability (T-AOC), total superoxide dismutase (T-SOD), glutathione peroxidase (GSH-Px), and malondialdehyde (MDA) in plasma were determined by following the manufacturer's instructions of the respective assay kits (Nanjing Jiancheng Bioengineering Institute, Nanjing, China).

Duodenum, jejunum, and ileum segments were removed, flushed with physiological saline to remove all the contents, and fixed in 4% paraformaldehyde. Three cross-sections were prepared for each sample after staining with hematoxylin and eosin using standard paraffin embedding procedures. The evaluated morphometric indices were as follows: villus height (Vh, from the tip of the villus to the crypt), crypt depth (Cd, from the base of the villus to the submucosa), and villus height-to-crypt depth ratio (Vh/Cd) (7). Morphological indices were measured using an image processing and analysis system (version 1, Leica Imaging System Ltd, Cambridge, UK). The mid-ileum (about 3 cm in length) of ileum was cut off and washed with an ice-cold isotonic saline buffer (pH 7), blotted with absorbent paper, then wrapped in aluminum foil, and stored at −80°C until the analysis of the immune indexes of the brush border membrane. The concentrations of sIgA, IL-2, IL-6, and TNF-α in the brush border membrane of the ileum were detected using commercially available enzyme-linked immunosorbent assay test kits (Shanghai Enzyme-Linked Biotechnology Co., Ltd., Shanghai, China).

### Statistical Analysis

Data were analyzed by one-way ANOVA using the PROC GLM procedure of the Statistical Analysis system, v 9.2 (SAS Inst. Inc., Cary, NC, USA). Orthogonal polynomials were applied for linear and quadratic effects of dependent variables to independent variables. Each replicate served as the experimental unit for all statistical analyses. Significant differences were set at *P* < 0.05.

## Results

### Growth Performance

Dietary full-fatted BSFM inclusion levels affected the final BW, ADG, and F/G and had no effect on ADFI and the mortality of layer chickens during 1–42 days of age (Table 2). As dietary full-fatted BSFM inclusion levels increased, there was a quadratic increase (*P* < 0.0001) in final BW and ADG and a quadratic decrease (*P* < 0.0001) in F/G (*P* = 0.0002). The chickens fed the diet with 3% full-fatted BSFM had the greatest ADFI and ADG as well as the lowest F/G during days 1 to 42.

**Table 2:** Effect of dietary low-inclusion levels of full-fatted black soldier fly larvae meal on the growth performance of layer chickens during 1–42 days of age (each value represents the mean of six replicates).

**Inclusion, %**	**Final BW, g**	**ADG, g/bird/day**	**ADFI, g/bird/day**	**F/G**	**Mortality, %**
0	467.1d	10.18d	22.73	2.23a	8.50
3	494.4a	10.83a	23.01	2.12c	6.67
6	481.4b	10.52b	22.46	2.14c	5.83
9	475.2c	10.37c	22.53	2.18b	6.67
SEM	1.95	0.047	0.175	0.011	1.23
*P*-value	<0.0001	<0.0001	0.15	<0.0001	0.63
Linear	0.21	0.16	0.21	0.003	0.45
Quadratic	<0.0001	<0.0001	0.56	<0.0001	0.24

*Final BW, final body weight; ADFI, average daily feed intake; ADG, average daily gain; F/G, feed/gain ratio; SEM, standard error of the mean*.

*Values within a column with no common letters differ significantly (P < 0.05)*.

### Nutrient Digestibility

Dietary full-fatted BSFM inclusion levels affected (*P* = 0.02) the apparent digestibility of CP and EE and had no effect (*P* > 0.11) on apparent digestibility in the DM, OM, Ca, and P of layer chickens at 42 days of age (Table 3). The digestibility of CP and EE was increased quadratically (*P* = 0.002) as dietary inclusion levels of full-fatted BSFM increased. The digestibility of CP and EE in chickens fed diets with 0 and 9% full-fatted BSFM was lower than that in birds fed a diet with 3% full-fatted BSFM.

**Table 3:** Effect of dietary low-inclusion levels of full-fatted black soldier fly larvae meal on apparent nutrient digestibility of layer chickens at 42 days of age (each value represents the mean of 6 replicates, %).

**Inclusion**	**DM**	**OM**	**CP**	**EE**	**Ca**	**P**
0	76.2	80.3	66.3b	81.3b	51.0	52.9
3	77.6	81.7	73.0a	84.5a	51.7	54.5
6	77.3	81.4	70.4ab	84.4a	51.1	52.5
9	74.0	78.0	67.0b	80.8b	50.8	51.4
SEM	1.44	1.12	1.37	0.94	1.34	1.08
*P*-value	0.31	0.12	0.01	0.01	0.97	0.28
Linear	0.30	0.18	0.97	0.67	0.82	0.20
Quadratic	0.12	0.05	0.002	0.002	0.72	0.24

*DM, dry matter; OM, organic matter; CP, crude protein; EE, ether extract; Ca, calcium; P, phosphorus*.

*Values within a column with no common letters differ significantly (P < 0.05)*.

### Intestinal Morphology

Dietary full-fatted BSFM inclusion levels had no effect (*P* > 0.05) on duodenum, jejunum, and ileum morphology, including Vh, Cd, and Vh/Cd (Table 4).

**Table 4:** Effect of dietary low-inclusion levels of full-fatted black soldier fly larvae meal on the intestinal morphology of layer chickens at 42 days of age (each value represents the mean of six replicates).

**Inclusion, %**	**Duodenum**			**Jejunum**			**Ileum**		
	**Vh, μm**	**Cd, μm**	**Vh/Cd**	**Vh, μm**	**Cd, μm**	**Vh/Cd**	**Vh, μm**	**Cd, μm**	**Vh/Cd**
0	921.2	90.4	10.12	739.6	106.5	6.86	464.9	96.4	4.88
3	926.3	83.4	11.10	776.2	133.0	6.04	528.0	117.9	4.62
6	1,000.1	87.1	11.48	812.1	122.9	6.67	487.7	122.9	3.99
9	934.6	88.2	11.56	796.3	128.4	6.28	509.0	114.2	4.51
SEM	50.2	5.2	0.67	25.4	8.0	0.35	24.8	7.53	0.29
*P*-value	0.45	0.55	0.69	0.26	0.06	0.36	0.33	0.10	0.21
Linear	0.24	0.65	0.24	0.09	0.10	0.56	0.18	0.06	0.22
Quadratic	0.15	0.53	0.83	0.32	0.16	0.80	0.21	0.03	0.22

*Vh, villus height; Cd, crypt depth; Vh/Cd, villus height-to-crypt depth ratio; SEM, standard error of the mean*.

### Ileum Mucosal Immunity

Dietary full-fatted BSFM inclusion levels had an effect (*P* < 0.03) on ileum mucosal sIgA concentration in layer chickens at 42 days of age (Table 5). No differences were observed in ileum mucosal IL-2, IL-6, and TNF-α concentrations in birds fed diets with different full-fatted BSFM inclusions (*P* > 0.03). An increase in dietary full-fatted BSFM inclusion levels resulted in linear and quadratic increases in ileum mucosal sIgA concentration.

**Table 5:** Effect of dietary low-inclusion levels of full-fatted black soldier fly larvae meal on ileum mucosal immunity of layer chickens at 42 days of age (each value represents the mean of six replicates).

**Inclusion, %**	**sIgA, ng/mL**	**IL-2, pg/mL**	**IL-6, pg/mL**	**TNF-α, pg/mL**
0	2,015b	150.3	15.12	66.5
3	2,202a	153.3	15.05	68.0
6	2,188a	152.7	15.80	66.1
9	2,184a	152.0	15.68	67.2
SEM	37	4.48	0.90	0.87
*P*-value	0.01	0.97	0.91	0.44
Linear	0.01	0.83	0.55	0.96
Quadratic	0.02	0.68	0.98	0.79

*sIgA, secretory immunoglobulin A; IL-2, interleukin-2; IL-6, interleukin-6; TNF-α, tumor necrosis factor alpha*.

*Values within a column with no common letters differ significantly (P < 0.05)*.

### Plasma Antioxidant Capacity

The dietary inclusion levels of full-fatted BSFM influenced (*P* < 0.02) the activities of GSH-Px and T-SOD as well as MDA content in the plasma of layer chickens at 42 days of age (Table 6). The activities of GSH-Px and T-SOD were increased linearly with the increase of full-fatted BSFM inclusion levels in the diet. The dietary inclusion of full-fatted BSFM at 6 and 9% levels had a higher plasma T-SOD activity than the diet with 3% full-fatted BSFM. An increase in dietary full-fatted BSFM inclusion levels resulted in a linear decrease in plasma MDA content in birds. The plasma T-AOC activity was not influenced (*P* > 0.21) by dietary full-fatted BSFM inclusion levels.

**Table 6:** Effect of dietary low-inclusion levels of full-fatted black soldier fly larvae meal on the plasma antioxidant capacity of layer chickens at 42 days of age (each value represents the mean of six replicates).

**Inclusion, %**	**T-AOC, U/mL**	**GSH-Px, U/mL**	**T-SOD, U/mL**	**MDA, nmol/mL**
0	7.89	410.5b	140.9ab	8.07a
3	8.28	476.8a	136.5b	6.33b
6	8.51	488.1a	148.3a	6.12b
9	8.49	476.8a	146.5a	6.06b
SEM	0.23	2.22	2.51	0.18
*P*-value	0.22	<0.0001	0.01	<0.0001
Linear	0.06	<0.0001	0.02	<0.0001
Quadratic	0.37	<0.0001	0.60	<0.0001

*T-AOC, total antioxidant capability; GSH-Px, glutathione peroxidase; T-SOD, total superoxide dismutase; MDA, malondialdehyde*.

*Values within a column with no common letters differ significantly (P < 0.05)*.

## Discussion

In recent years, protein of insect origins, such as BSFM, has received considerable attention as sustainable alternatives to conventional protein sources (fish or plant protein meals) used in poultry (2, 10). The potential use of BSF as a promising insect species that is able to replace dietary fish meal and soybean meal either partially or completely has already been evaluated in poultry. For layers, two recent studies found that using defatted BSF larvae as feed source can increase egg quality, such as eggshell thickness, egg yolk, and egg albumin, in hens at the laying period (4, 22). However, little information was available for layer chickens at the starter growth period. The starter brooding period is critical for the development of growth, bone, and gastrointestinal tract to minimize the mortality and keep the uniformity of young laying hens, which could directly affect the later egg production performance of laying hens. In the present study, as dietary full-fatted BSFM inclusion levels increased, final BW and ADG were increased quadratically in layer chickens during 1–42 days of age. The greatest final BW at day 42 and ADG during days 1–42 were observed in birds fed the diet with 3% full-fatted BSFM inclusion level. The positive effects of full-fatted BSFM on growth performance were consistent with those reported in broilers (23) and broiler quails (24) fed diets with the inclusion of defatted BSFM. On the one hand, compared with plan protein sources, full-fatted BSFM is characterized by the balanced amino acid composition and mono- and poly-unsaturated fatty acid profiles, which can be better utilized to feed poultry. Additionally, some studies have demonstrated that the dietary incorporation of full-fatted BSFM improved meat quality *via* modifying the fatty acid profile in pigs (11, 12). On the other hand, substituting the plant protein of soybean meal and corn gluten meal with BSFM in basal diet could further increase nutrient digestibility by reducing the content of plant-derived anti-nutritional substances (i.e., non-starch polysaccharide), which can also explain the benefit of dietary BSFM inclusion on growth performance. However, in contrast to other previous findings of birds (7), the results showed that a quadratic decrease of final BW and ADG was observed in ducks during 39–50 days of age when the dietary inclusion levels of defatted BSFM increased from 0 to 9%. Ewald et al. (15) demonstrated that increasing levels of defatted BSF meal resulted in decreasing feed consumption and final BW of broiler chickens. Other reports showed that a defatted BSF meal could be introduced in the diet, partially replacing conventional soybean meal and soybean oil, with no negative effects on growth performance and carcass traits for growing broiler quails (24) and broiler chickens (23). These inconsistent results could be due to differences in poultry species and age, insect species and the substrates, and processing techniques (defatted BSFM vs. full-fat BSFM).

The apparent nutrient digestibility reflects the degree of absorption and utilization of the dietary nutrient. Defatted BSF meals have been evaluated as an excellent source of apparent metabolizable energy and ileal amino acid digestibility in poultry (9). This more efficient nutrient digestion suggested the effective utilization of defatted BSFM in poultry feed formulation (10). Therefore, there is an urgent need to explore the nutritional value of full-fatted BSFM, which is comparable to that of defatted BSFM or other animal protein sources, for potential use in poultry diets. In the present study, as with the increased dietary inclusion levels of full-fatted BSFM, a quadratic increase in the digestibility of CP and EE was observed in layer chickens at 42 days of age. The digestibility of CP and EE in chickens fed diets with 3% full-fatted BSFM was higher than that in birds fed the control diet. The improvement in EE digestibility agreed with the results observed in ducks during days 18–38, following the increasing inclusion levels of defatted BSFM (7). A recent study revealed that the fat derived from BSFM is rich in medium-chain fatty acids that can have a positive effect on increasing the energy availability in the intestine (17, 25), thus promoting growth performance. However, full-fatted BSFM meal included in duck diets by as much as 9% displayed an inhibited effect on the digestibility of CP and EE of chickens at 42 days of age. Similarly, a significant reduction in the rate and the efficiency of protein deposition was observed in trout fed a diet at 13.2% inclusion of defatted BSFM and beyond (13). It was also proven that CP digestibility of BSFM *in vitro* was negatively correlated to its chitin content (21). Therefore, the chitin content supplied by the BSFM may have been responsible for the impaired fat and protein digestibility in the intestinal tract of broiler chickens at high inclusion levels of BSFM (26). However, dietary BSFM inclusion had no positive influence on the gut morphology and the histological findings of the layer chickens in our study. This is in line with previous findings in ducks (7) and weanling piglets (11). It is hypothesized that BSFM may have a beneficial effect on nutrient absorption by affecting other aspects of gut health rather than intestinal morphology.

The maintenance of proper functioning and health of the gastrointestinal tract is crucial for ensuring an adequate growth performance in poultry. The feed ingredients and nutrients can affect the intestinal function of the host (27). In the current study, the increase of full-fatted BSFM inclusion levels in diets resulted in linear and quadratic increases of sIgA level in the ileum mucosa of birds at day 42. Dietary supplementation with full-fatted BSFM increased the serum IgA and IL-10 levels of the anti-inflammatory cytokine IL-10 and immunoglobulin IgA as well as decreased the level of pro-inflammatory cytokine IFN-γ (12). These positive results implied that the replacement of BSFM may have a beneficial effect on immune homeostasis in the host. In addition, BSFM increases the survivability of broiler chicks against an experimental infection of *Salmonella gallinarum via* enhancing immune activities (28). In terms of BSFM, a proportion of lauric acid and antioxidant peptide was characterized by important antioxidant ability and antibacterial activity. For example, previous studies indicated that lauric acid could enhance intestinal immune ability due to its antioxidant and prebiotic properties (16, 29). In the present study, dietary full-fatted BSFM inclusion enhanced the antioxidant capacity with the linear increase of GSH-Px and T-SOD activities in serum. MDA, as an indicator of endogenous oxidative damage, presents a final product of lipid peroxidation (30). In our study, the serum MDA content was decreased as with the increase of full-fatted BSFM inclusion due to the stronger activity of antioxidant enzymes in the defense system. Similar encouraging results have been confirmed in several fish species (31, 32). It has been reported that antioxidant ability due to chitin appears to have a positive effect on the immune system of poultry, as it exhibits prebiotic properties in the large intestine and appears to exhibit a bacteriostatic effect on Gram-negative bacteria (33).

## Conclusions

A range of dietary full-fatted BSFM inclusion levels increased quadratically the final BW and ADG as well as the digestibility of CP and EE in layer chickens during 1–42 days of age. In addition, dietary full-fatted BSFM inclusion levels could increase linearly the plasma GSH-Px and T-SOD activities and ileum mucosal sIgA, resulting in greater antioxidant capacity and ileum mucosal immunity in layer chickens at 42 days of age. The encouraging results of the improvement of growth performance and gut health parameters suggested that partially full-fatted BSFM inclusion can be suitable protein ingredients for layer chickens' diets at the starter period.

## Data Availability Statement

The original contributions presented in the study are included in the article/Supplementary Materials, further inquiries can be directed to the corresponding author/s.

## Ethics Statement

The experimental protocol was reviewed and approved by the Animal Care and Use Committee of Liao Cheng University.

## Author Contributions

XC and LL designed this study, carried out the experiments and measurements, and drafted the manuscript. ML wrote (review and editing) the manuscript and analyzed the data. GW helped with the sample analysis. RS and ZW assisted with the trial. KW helped with the data analysis. LL and KW participated in the study design, coordination, and manuscript writing. All the authors read and approved the final manuscript. All authors contributed to the article and approved the submitted version.

## Conflict of Interest

The authors declare that the research was conducted in the absence of any commercial or financial relationships that could be construed as a potential conflict of interest.

## Abbreviations

BW: body weight
ADFI: average daily feed intake
ADG: average daily gain
F/G: feed/gain ratio
Vh: villus height
Cd: crypt depth
Vh/Cd: villus height-to-crypt depth ratio
T-AOC: total antioxidant capability
T-SOD: total superoxide dismutase
GSH-Px: glutathione peroxidase
CAT: catalase
MDA: malondialdehyde
DM: dry matter
OM: organic matter
CP: crude protein
EE: ether extract
Ca: calcium
P: phosphorus
sIgA: secretory immunoglobulin A
IL-2: interleukin-2
IL-6: interleukin-6
TNF-α: tumor necrosis factor alpha.
